# MHC class II of different non-professional antigen-presenting cells mediate multiple effects of crosstalk with CD4^+^T cells in lung diseases

**DOI:** 10.3389/fmed.2025.1388814

**Published:** 2025-01-17

**Authors:** Ming-Yan Wang, Yu Qiao, Shan-Jie Wei, Zhao-Liang Su, Hong-Yan Lu

**Affiliations:** ^1^Department of Pediatrics, The Affiliated Hospital of Jiangsu University, Zhenjiang, China; ^2^International Genome Center, Jiangsu University, Zhenjiang, China; ^3^Institute for Medical Immunology, Jiangsu University, Zhenjiang, China

**Keywords:** alveolar epithelial cells, major histocompatibility complex, antigen-presenting cells, lung disease, T cells

## Abstract

The respiratory system is continuously exposed to the outside world, making it vulnerable to airborne particles and harmful pathogens like bacteria and viruses that can enter through breathing. Antigen presenting cells (APCs) have a vital function in the innate immune response as they present antigens to T cells and initiate the response of adaptive immune cells. Professional APCs engulf foreign microorganisms and display their peptides to T lymphocytes using MHC molecules. MHC II on their cell surface and potentially present antigen to CD4^+^T cells. Furthermore, various other types of cells have similar function that can also serve as APCs by expressing MHC II, thus impacting the progression of lung diseases, such as alveolar epithelial cells (AECs), endothelial cells (ECs), fibroblasts, innate lymphoid cells (ILCs), eosinophils, interstitial cells, mast cells, etc. express MHC II and present antigen. The non-professional APCs type and the extra signals it provides have a direct impact on CD4^+^T cell programming and downstream effector mechanisms. Here, we summarize the existing research on the expression of MHC II on non-professional APCs in different lung diseases and its influence on CD4^+^T differentiation types and disease outcomes, in order to further clarify the role of MHC II of different non-professional APCs in lung diseases, such as asthma, chronic obstructive pulmonary disease (COPD), etc.

## 1 Introduction

Professional Antigen presenting cells (APCs) in the lung, such Dendritic cells (DCs), macrophages (MACs), and B cells, come into contact with T cells that are specific to the antigen and have a crucial and distinct function in promoting interaction between the innate and adaptive immune systems, guaranteeing defensive immunity and creating immune memory. When encountering foreign pathogens, APCs activate naïve T cells through antigenic peptides and signals (1). The interaction leads to the stimulation of T cell differentiation and proliferation, resulting in the formation of long-lasting memory T cell populations like central memory T (TCM) cells, effector memory T (TEM) cells, and resident memory T (TRM) cells (2–4). Earlier research has recognized separate groups of APCs in the respiratory system, where the interaction between APCs and T cells heavily relies on major histocompatibility complex (MHC) molecules. T lymphocytes recognize antigens through the presence of MHC molecules on the surface of APCs. MHC molecules responsible for displaying antigens comprise MHC class I, MHC class II, and MHC-like CD1 molecules (5).

CD4^+^T cells play an important role in lung disease, showing a high degree of heterogeneity and plasticity, and are regulated in response to stimulation with cognate antigens bound to MHC class II molecules. Thus, the antigen-presenting cell type and the additional signals it provides directly affect CD4^+^T cell programming and its downstream effector mechanisms. The expression of MHC II is mainly limited to professional APCs, but epithelial cells and fibroblasts can also express MHC II. However, the functional consequences of CD4^+^T cell interactions with these “non-professional” APCs are unknown.

Additionally, it has been proposed that various cell types in the lung, including alveolar epithelial cells (AECs), endothelial cells (ECs), innate Lymphoid Cells (ILCs) and eosinophils in the lung, may have the ability to present exogenous antigens to T cells (6, 7). The cell categories are commonly known as non-professional APCs. APCs load foreign antigen-derived peptides onto MHC II, which then present peptide–MHC II complexes to CD4^+^T cells (8–11). It is interesting to note that approximately half of the cells expressing MHC II in the lung are CD45-nonhematopoietic cells (12).

Mounting evidence suggests that non-hematopoietic cell populations residing in tissues play a crucial role in controlling immune responses specific to organs (13). During perinatal development, AECs progenitors undergo a process of differentiation into mature alveolar type 1 (AEC I) and type 2 (AEC II) cells. According to recent findings, it has been indicated that additional cell types in the respiratory system, like AEC II, possess the ability to present antigens (14–16).

The literature on immune signaling and immune cellular changes in lung inflammatory diseases has experienced significant growth in recent years. Currently, there have been studies on the impact of non-immune cells presenting antigens on the onset and progression of various illnesses. Non-professional APCs have gradually become the focus of research. In the realm of cardiovascular and other disease research, the discovery of the involvement of non-professional APCs in disease is an ongoing process. A recent report in Nature revealed that cardiac fibroblasts play a role in promoting cardiac fibrosis and dysfunction through antigen presentation (17).

While professional APCs in the lung are well understood in their role of activating T cells and generating immune memory, the functional implications of interactions between non-professional APCs and CD4^+^T cells remain unclear. CD4^+^T cells are activated by other lung cells such as AECs, ECs, fibroblasts, innate lymphoid cells (ILCs), eosinophils, interstitial cells, mast cells, etc. through antigen presentation. Specifically, the ability of these non-professional APCs to influence T cell differentiation, programming, and effector functions, as well as their contribution to immune responses in lung diseases, is not fully elucidated. The main emphasis of this review focuses on the expression of MHC II on non-professional APCs cell surface and potentially present antigen to CD4^+^T cells in lung development and different lung diseases. In detail, we summarize the expression of MHC II on non-professional APCs and CD4^+^T cells formation by non-professional APCs during lung diseases, such as asthma, chronic obstructive pulmonary disease (COPD), etc. This analysis will help to understand the significance of MHC II mediate multiple effects of crosstalk with CD4^+^T cells in lung diseases.

## 2 How to identify a cell as an antigen presenting cell

In order to comprehend the reason behind the ability of specific non-immune cells to function as APCs, as well as the correlation between their function and lung diseases, it is imperative to initially grasp the attributes of APCs. To start with, it is necessary to possess the capacity to modify the proteasome, such as through the activation of the pertinent genes that stimulate the proteasome, as these immunoproteasomes play a role in the processing of antigenic peptide (18). Additionally, in order to present antigens to CD8^+^T or CD4^+^T cells, APCs must express either MHC I or MHC II, respectively. It is necessary for cells to have the capability of increasing the expression of co-stimulatory proteins like B7, which interacts with CD28 found on the outer layer of T cells (19). Successful activation of T cells necessitates this interaction. Activation of antigen-dependent T cells necessitates the presence of these three essential functions in APCs. While these genes can be expressed by various cell types and their expression can increase in response to disease or stimulation, the upregulation of these genes alone is not enough to ascribe APC function. Additionally, it must be demonstrated that APCs are effective and capable of inducing T cell activation. In short, the most direct way to identify a cell as an APC is to test whether it expresses MHC I and MHC II molecules. The expression of costimulatory molecules (such as CD80, CD86, etc.) was examined. To verify whether it has the ability to uptake and process antigens. The effective activation of specific T cells was verified by co-culture assay or T cell proliferation/activation assay. With these methods, can effectively determine whether a cell has antigen-presenting function.

## 3 Evidence for the formation of crosstalk between non-professional APCs and CD4^+^T cells via MHC II in lung diseases

The cellular contribution of the lung to the adaptive immune response is emerging but not fully understood. New findings indicates that there may be significant functional connections between T cells within the lungs and non-professional APCs, which could potentially contribute to development of diseases and lung-related issues. The results reveal that cells like the AECs function as the primary APCs in the lung and carry significant implications for disease comprehension.

Pulmonary immunity is significantly influenced by CD4^+^T cells. Furthermore, their traditional role in aiding the initiation of CD4^+^T cell activation in lymph nodes is also noteworthy. In the lung, CD4^+^T cells are also responsible for regulating both proinflammatory and anti-inflammatory immune responses. In addition, CD4^+^T cells have the ability to express cytotoxic programs that are comparable to those observed in CD8^+^T cells. CD4^+^T cells display a significant amount of diversity and flexibility and are controlled when exposed to MHC II-bound specific antigens. Hence, the specific cell category displaying the antigen and the supplementary cues it offers have a direct impact on the programming of CD4^+^T cells and subsequent effector mechanisms. While MHC II expression is primarily limited to APCs, there are certain situations where MHC II can also be expressed. Nevertheless, the functional implications of intrapulmonary interactions between CD4^+^T cells and this particular non-professional APCs (AECs, ECs, fibroblasts, ILCs, eosinophils, interstitial cells, and mast cells) are currently being examined.

The lung contains several non-professional APCs that also have the ability to present antigens. These cells induce the expression of MHC II molecules and possess a limited capacity for antigen presentation. The non-professional APCs found in the lung include AECs, ECs, fibroblasts, ILCs, Eosinophils, Interstitial cells, and Mast Cells.

In the upcoming parts of this analysis, we will gather the newly gathered compelling proof that certain non-immune cells in the respiratory organ can carry out numerous of these tasks and can thus can function as APCs in particular situations or contribute to the disease procession.

### 3.1 Alveolar epithelial cells

**Figure 1:**
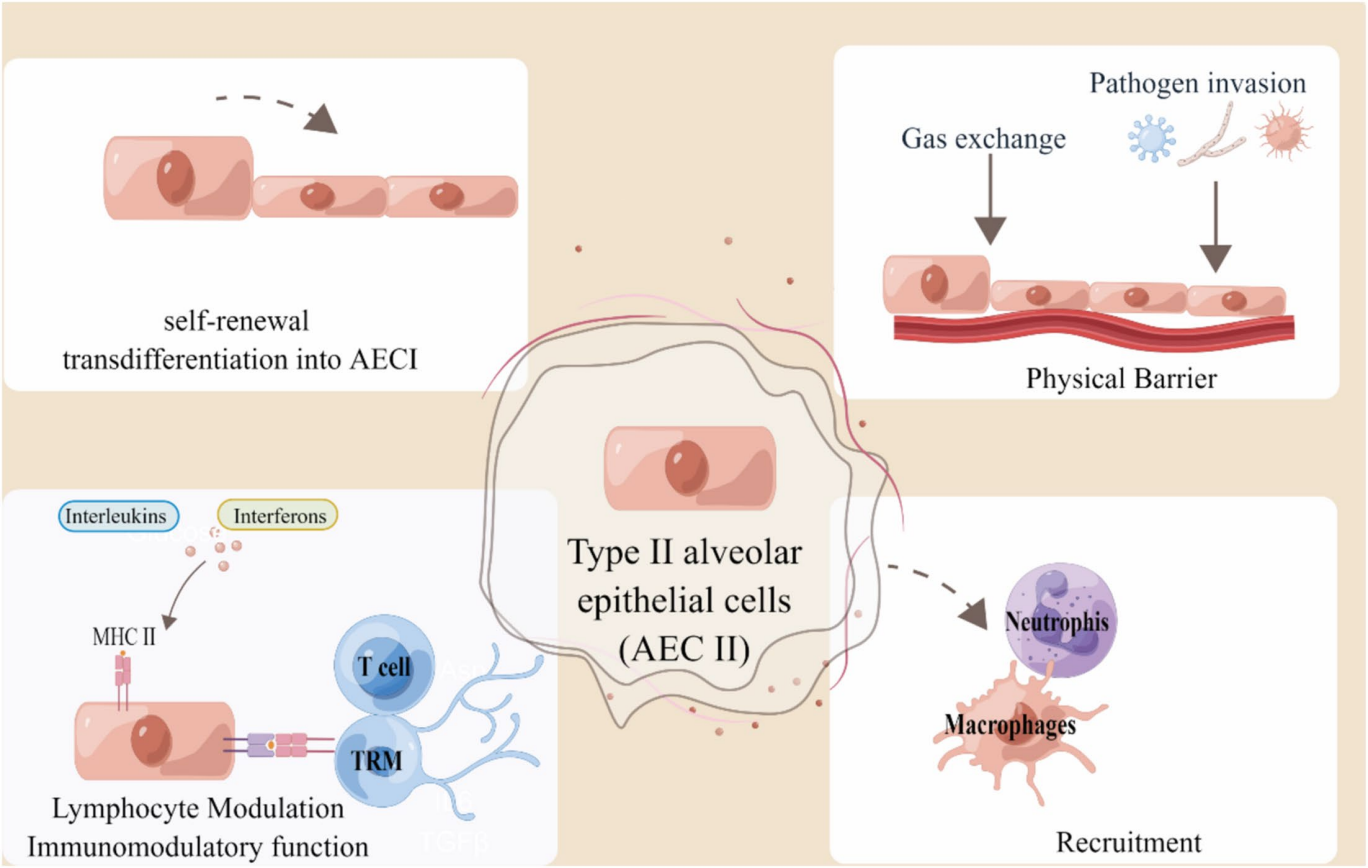
Alveolar type II epithelial cells in lung immunity. AEC II serves as an external obstacle and activates the innate immune response to engage in immune modulation. Type II epithelial cells can act as non-professional APCs by presenting antigen to T cells and TRM cells in a manner restricted by MHC II, thereby indicating their involvement in the regulation of immune function. The figure was created using Figdraw (www.figdraw.com).

AECs consist of epithelial cells, namely AEC I and AEC II (20). AEC I have the primary responsible of facilitating gas exchange and also play a crucial role in the regulation and defense mechanisms (21). As progenitor cells, AEC II have the ability to either self-renew or differentiate into AEC I cells, primarily generating surfactant (21, 22). Simultaneously, they have the ability to generate cytokines and chemokines when faced with different types of lung damage caused by bacteria, viruses, or mechanical ventilation, and also play a role in immune reactions (23). AECs have direct interactions with the surrounding environment, making them crucial elements in the regulation of barrier immunity (13). AEC II has a barrier function to prevent external pathogens and harmful substances from entering the lung tissue. In the case of infection or injury, it secrete cytokines, activates the immune system, attracts and recruits immune cells to the inflammatory site, and also provides antigen information to T cells through the expression of MHC molecules to play a local antigen presentation role. When injury, occurs AEC II differentiates to AEC I to ensure renewal and homeostasis. Here, we summarize the functional role of AEC II in pulmonary immunity in Figure 1.

#### 3.1.1 MHC II expression levels on AEC II during lung development

Decades ago, it was demonstrated that AECs express MHC II. However, the precise role of this expression is still not understood. Currently, there has been extensive research on AECs, which are increasingly acknowledged for their role in maintaining immune balance. Increasing evidence suggests that alveolar epithelial cells play a role in the lung’s adaptive immune response (21). AECs have the ability to express MHC II protein and associated processing molecules, potentially serving as non-professional APCs (24).

Historically, DCs, MACs, and B cells have been categorized as professional APCs and would normally express MHC II. Nevertheless, numerous researches have detected the presence of MHC II expression in lung epithelial cells under normal conditions and in reaction to viral damage or cancer (25, 26). It is worth noting that the presence of invariant chain expression has been observed on fetal alveolar epithelium in humans at 12–14 weeks gestational age, even in the absence of MHC II co-expression (27). During gestation, it seems that MHC II is not expressed on the surfaces of AECs in fetal lung tissue, unless there is active inflammation (28). Initially, it was demonstrated that adult AECs consistently express MHC II on the bronchial and alveolar epithelium, particularly on AEC II and ciliated ECs (29).

There is limited research on the role of AEC II expressing MHC II during lung development. Studies have shown that immune control in the lung is mediated through the expression of MHC II by pulmonary epithelial cells. These cells act as sentinels with anti-infection capabilities. In cell culture, pulmonary epithelial cells use these molecules to instruct T cells on their actions. This allows them to respond appropriately to infectious microorganisms, suggesting that AEC II may play a role in infection monitoring during lung development through antigen presentation.

#### 3.1.2 The multiple effect was mediated by AEC II antigen-presenting CD4^+^T cells in lung disease

There is increasing evidence that AEC II in the lung contribute to adaptive immune responses in the lungs. AEC II cells express MHC II and present antigen. In lung infectious diseases, Toulmin et al. showed that AEC II had antigen presentation ability, but it was lower than DCs (25). *In vitro* antigen-presenting experiments AEC II with antigen specific hybridoma, they revealed that while AEC II can present antigens to CD4^+^T cells and induce interferon-*γ* (IFN-γ) production, the efficiency is lower than DCs, especially in the context of influenza A virus (IAV) infection. The absence of MHC II on AEC II cells led to a slight deterioration in respiratory viral illness after being infected with influenza and Sendai virus (25, 30). The presence of IFN-*γ* in the lung microenvironment has the ability to control the presentation of different antigens and the subsequent interactions between T cells and non-professional APCs (31). The presentation of antigens by AEC II cells could play a crucial role in viral infections by enabling a proficient group of APCs to quickly obtain antigens from infected epithelial cells. For instance, immune cells can receive MHC II from epithelial cells (32).

SPC^low^MHC II^high^ AEC II cells act as APCs to generate CD4^+^TRM cells (33). *In vitro*, AEC II cells that present antigens stimulate naïve CD4^+^T cells and promote the development of regulatory T cells (34). Barrier epithelial cells not only activate CD4^+^T cells but also attract and retain CD8^+^TRM cells in close proximity to the sites of antigen exposure and reactivate them through local antigen presentation (2, 35). Recent studies have provided fresh insights into the interactions between epithelial cells and CD4^+^TRM cells (33).

Research conducted on mice with a targeted elimination of MHC II in AEC II has shown the significance of MHC II in relation to these cells. The absence of MHC II on AEC II cells resulted in a decrease in the quantity of T cells in the lungs and the percentage of CD4^+^T cells that exhibited PD-1 (35). In a more striking manner, animals that do not have MHC II in their epithelial cells experience a mortality rate that is twice as high as that of control animals after IAV infection (25). This emphasizes the benefit of survival provided by MHC II^+^ AEC II cells. Interestingly, Shenoy and colleagues discovered that the targeted removal of MHC II in lung epithelial cells led to a decrease in PD-L1 expression by AECs, both in normal conditions and in pneumonia caused by bacteria (33). After being re-infected, AEC II cells that present antigens may reactivate effector T cells or start the recruitment of regulatory T cells in order to support the regeneration of epithelial cells (36). CD4 ^+^TRM cell maintenance around airways was facilitated by airway epithelial cells in a model of *S. pneumoniae* infection (33). The suppression of CD4^+^T cells may occur due to the inhibition of overly exuberant and potentially harmful responses, which is caused by the expression of PD-L1 in epithelial cells (37).

AECs from individuals with allergy or autoimmunity, such as chronic bronchitis, asthma, idiopathic pulmonary fibrosis (IPF), or lung transplant rejection, exhibit increased MHC II expression in lung tissue (30, 38–40). Increased MHC II expression on AEC II is a phenomenon of interest in these lung diseases. The increase in MHC II may be related to bystander effect during any chronic inflammatory condition, but the current understanding of its specific mechanisms and effects is still incomplete. This effect may modulate the local immune environment or promote inflammatory responses. However, there is no direct evidence that upregulation of MHC II plays a direct antigen presentation role in these diseases. Although the increase in MHC II on AEC II suggested that these cells may have potential function for antigen presentation, no study has clearly demonstrated efficient antigen presentation by MHC II on AEC II in these lung diseases. In IPF and lung transplant rejection, the increased expression of MHC II on AEC II may be related to the fibrotic process of the disease and immune rejection, but its specific role in antigen presentation needs to be further explored. A summary of the expression of these MHC II-expressing non-professional APCs and their detection methods is important for further investigation of the function of these non-professional APCs. Based on this, we summarize the current relevant *in vitro* and *in vivo* studies on the expression and outcome of MHC II molecules on AECs in lung disease shown in Table 1.

**Table 1:** *In vitro* and *in vivo* studies of MHC II expression in AECs in different lung diseases.

Authors	Species	Disease type	Technique	Assays/cell lines	Outcome/findings
Peters et al. (28)	Human	Intrauterine infection	Correlational study	IHC	Pulmonary inflammation is closely related to the expression of MHC II molecular products on lung epithelial cells
Cunningham et al. (24)	Human	Normal	In vitro, IFN-γ stimulation of MHC II expression	Cell line	Expression of MHC II can be induced by stimulation with the proinflammatory cytokine IFN-γ
Shenoy et al. (33)	Mouse	*Streptococcus pneumoniae* (Spn)	MHC II gene was specifically knocked out in lung epithelium	Histology	MHC II expression was high on AEC II and SPC^low^MHC^high^ LECs
Kallenberg et al. (65)	Human	Patients with fibrosing alveolitis	Correlational study	IHC	AECs were positive for HLA-DR monoclonal antibody
Kaneko et al. (40)	Human	Interstitial lung diseases	Correlational study	IHC	Abnormal expression of MHC II
Vignola et al. (66)	Human	Asthma	Correlational study	immunocytochemistry	HLA-DR is significantly increased in epithelial cells of asthma patients
Dias et al. (67)	Human	Live *Mycobacterium leprae* infection	Correlational study	A549 cell line	*M. leprae* can induce MHC II expression
Guo et al. (68)	Human	Inflammation-driven lung adenocarcinoma (IDLA)	In vitro, TNF-α stimulation of MHC II expression	IHC	AEC II cell-derived tumor cells lead to Treg expansion in an MHC II-dependent manner
Shen et al. (69)	Human	Aflatoxin G1 (AFG1) induces inflammation-induced lung cancer	In vitro, TNF-α and Aflatoxin G1 (AFG1) stimulation of MHC II expression	A549 cell line primary human AEC II cells	Synergize to enhance MHC II
Hasegawa et al. (70)	Mouse	Normal	Correlational study	Immunofluorescence	The purity of isolated AEC II was improved by EpCAM and MHC II expression
Ibrahim et al. (71)	Human	CMV infection	Infected cells were incubated with IFN-γ	IHC	The presence of IFN-γ produced by infiltrating activated monocytes in the microenvironment of the transplanted lung allows lung epithelial cells to act as antigen presenting cells and express high levels of MHC II

### 3.2 Endothelial cells

The lung has a high amount of blood vessels due to a unique arrangement of endothelial cells, primarily made up of microvascular endothelial cells. Capillaries, which are small blood vessels, closely interact with the alveoli to enable the exchange of gasses between the apical circulation and the air in the basal alveoli (41). The pulmonary ECs are the cells that line the inner surface of these capillaries, Immune-cell recruitment primarily takes place in the lung’s capillaries, which are usually just a few micrometers wide. Due to the narrowing of capillaries, recruited immune cells tend to slow down as they traverse these vessels, resulting in a longer contact time between immune cells and ECs to potentially modulate the immune response. T cells must pass through these cells, which compose the inner lining of blood vessels, in order to reach their target, thus establishing a direct barrier between blood and tissue (42). Hence, ECs have the ability to effectively display antigens derived from blood and neighboring tissues to circulating T cells. Krupnick AS et al. showed that the activation and induction of CD4^+^Foxp3^+^ regulatory T cells occur when antigens are presented to CD4^+^T cells through the vascular endothelium (43). Infection of lung ECs, such as capillary ECs, by IAV, could potentially trigger the presentation of viral antigens and aid in the prompt activation of memory T cells located within blood vessels or surrounding tissues (44).

### 3.3 Fibroblasts

Fibroblasts frequently reside near lung epithelial cells and have demonstrated an ability to present antigens (17, 45). Several experiments conducted in a laboratory setting using mice have shown that fibroblasts can effectively display antigens to CD4^+^T cells (17, 46). Ngwenyama et al. studied the interactions between fibroblasts and CD4^+^T cells in a heart failure mouse model. They found that cardiac fibroblasts present antigens to CD4^+^T cells through MHC II, induced by IFN-*γ*. In the lung, recent single-cell RNA sequencing studies have further shed light on fibroblasts, previously thought to be uniformly supportive, for example also having specific pathogenic properties such as antigen presentation and vascular support. Antigen presentation by lung MHC II^+^fibroblasts is a crucial driver of anti-tumor immunity. The MHC^+^fibroblasts, known as antigen-presenting cancer-associated fibroblasts (apCAFs), present cancer-specific MHC II peptides to nearby CD4^+^T cells. In a recent study, Kerdidani and colleagues discovered MHC II^+^ lung fibroblasts in both normal conditions and in mouse models of lung cancer, which were found adjacent to regions with a high density of CD4^+^T cells (26). They demonstrated that cancer-specific CD4^+^T cells exhibited increased expression of effector cytokines when stimulated by apCAFs. This interaction enhances the effector function of cancer-specific CD4^+^T cells and contributes to the tumor-suppressive effect of apCAFs. Lung antigen-presenting fibroblasts may be unique in their ability to activate effector T cells. Denton et al. discovered lung fibroblasts in the lung infected with IAV that facilitated the development of lung germinal centers by encouraging the recruitment of B cells (47). The fibroblasts in the naïve lung were not detected and expressed MHC II. The findings indicate that MHC II^+^ lung fibroblasts could potentially interact with lung T cells that have encountered antigens, thereby enhancing immune responses that provide protection (48).

HLA-DR and costimulatory molecules OX-40 L and CD70 are expressed by human lung fibroblasts, enabling the activation and maintenance of CD4^+^ memory T cells (49). Overall, MHC II expression in lung fibroblasts may play an important role in regulating local immune responses, disease progression, and immune balance.

### 3.4 Innate lymphoid cells

ILC2s, also known as Type 2 innate lymphocytes, play a crucial role in initiating early immune responses associated with type 2 immunity (50). Previous research has indicated that ILC2s are capable of expressing MHC II molecules, thus functioning as antigen-presenting cells (APCs) (51). ILC2s strongly express OX40L, ICOS, ICOSL, CD80, and CD86, which have the ability to control the differentiation of CD4^+^T cells (52).

ILC2s isolated from the peripheral blood of patients experiencing acute exacerbation of chronic obstructive pulmonary disease (AECOPD) were co-cultured with CD4^+^T cells obtained from the peripheral blood of healthy individuals. This co-culture aimed to investigate the potential role of ILC2s as APCs by upregulating MHC II and influencing the shift toward Th2-type responses in AECOPD (53). In their study, Kang and colleagues showed that pulmonary ILC2s have the ability to function as APCs, triggering CD4^+^T cell activation through the MHC II pathway in the context of respiratory syncytial virus (RSV) infection (54). The presence of RSV infection enhances the presentation of MHC II molecules on the outer layer of pulmonary ILC2s. ILC2s have the ability to trigger and facilitate the growth and specialization of CD4^+^T cells infected with respiratory syncytial virus. Significantly decreasing the expansion of CD4^+^T cells was achieved by blocking the interaction between CD4^+^T cells and ILC2s using a monoclonal antibody against MHC II.

Furthermore, ILC3s have the ability to directly control CD4^+^T cells by presenting antigens to MHC II (55–57). The study by Teng et al. found that antigen-presenting ILC3s play a crucial role by expressing a high amount of MHC II and effectively restricting the growth of allergen-specific CD4^+^T cells during airway challenge in experimental airway inflammation (58).

### 3.5 Other non-professional APCs to consider: eosinophils, interstitial cells, mast cells

Hansel et al. reported that eosinophils expressed HLA-DR at sites of active disease processes (59). In addition, they have demonstrated that eosinophils are involved in antigen uptake, processing and presentation, and HLA-DR^+^eosinophils can present antigen to CD4^+^T lymphocytes. Additional confirmation was provided by the findings of Beninati W et al., which detected eosinophil HLA-DR expression at sites of human disease (60). This molecule was identified on eosinophils isolated from bronchoalveolar lavage (BAL) of patients with asthma and chronic eosinophilic pneumonia. This study primarily identified that mature eosinophils can express MHC II molecules, indicating a potential immunological function. Further research has shown that eosinophils in asthma patients and experimental models, it has been demonstrated that eosinophils express MHC II and costimulatory molecules that are crucial for APCs (61). The study conducted by Zhang and colleagues showed that eosinophils derived from mice infected with RSV can function as APCs and are responsible for triggering asthma (62).

Interstitial cells containing I-A-like MHC II were observed in the trachea and lungs of both conventional specific pathogen free (SPF) and germ-free rats (30). Nakano et al. found that mast cell expression of MHC II and OX40L is induced to increase through Notch signaling, thereby inducing a preferential shift of naïve CD4^+^T cells to Th2 cells with an antigen-presenting function and may play a role in allergic diseases (63).

In conclusion, the diverse roles of non-immune cells with antigen-presenting capabilities in the lung underscore their complex contributions to pulmonary diseases. While cells such as fibroblasts, ECs, eosinophils and mast cells can exhibit antigen-presenting functions, their effects vary significantly depending on their specific context and interactions within the lung microenvironment. These variations in antigen presentation not only influence the immune response but also impact the progression and outcome of pulmonary conditions.

## 4 Potential clinical translation of non-professional APCs in pulmonary diseases

The immune system, including the adaptive immune system, is considered to be the key to promote the regenerative response after injury (51). Recent studies have shown that the antigen pressing-adaptive immune axis plays a role in zebrafish heart regeneration, but this has not been studied in lung regeneration, which may be the light of future research on lung regeneration (64). The potential clinical transformation of non-professional APCs in pulmonary diseases has increasingly become a research focus. Recent studies highlight the significant role these cells may play in early diagnosis, therapeutic target development, and prognosis evaluation. Therefore, further exploration of non-professional APCs in pulmonary diseases is crucial for their clinical application.

Non-professional APCs express MHC II and costimulatory molecules under specific conditions. This enables them to perform antigen presentation functions in pulmonary diseases. These cells are integral to the local immune response by activating CD4^+^T cells. Understanding these functional changes in pathological environments helps elucidate disease mechanisms and offers new clinical insights.

From a clinical perspective, the expression patterns of non-professional APCs in pulmonary diseases present opportunities for early diagnosis. Monitoring MHC II and related molecule expression levels could help detect abnormal immune responses early, allowing for timely intervention. Additionally, the antigen-presenting ability of non-professional APCs at various disease stages may serve as biomarkers for disease progression and prognosis. For example, increased MHC II expression in fibroblasts during early pulmonary fibrosis could indicate immune dysregulation and tissue remodeling, aiding early diagnosis and disease classification.

Since COPD and IPF may often be multifactorial diseases, non-professional APCs may interact with other immune cells and factors in the pulmonary microenvironment. They not only play a key role in the immune response, but also may aggravate disease progression through the promotion of fibrosis. Although clinical translational research faces some challenges, it may become a new strategy for the treatment of COPD and IPF by regulating the function of these cells. Therefore, future treatment may require a multi-target strategy that combines the regulation of non-professional APCs with the intervention of other immune mechanisms to effectively improve the prognosis of patients. In-depth study of the specific role and mechanism of non-professional APCs in these diseases could help to develop new therapeutic strategies and alleviate the long-term effects of the disease. Moreover, non-professional APCs show potential as therapeutic targets in pulmonary diseases. Modulating their antigen-presenting functions could influence the immunopathological processes. For instance, inhibiting MHC II expression or costimulatory signaling in lung fibroblasts might reduce chronic inflammation and fibrosis. Immunomodulatory strategies targeting these cells could also benefit conditions like COPD, IPF and asthma by mitigating inflammatory responses and slowing disease progression. However, translating non-professional APCs research into clinical practice presents challenges. The functional heterogeneity of these cells across different diseases and microenvironments complicates targeted regulation. Non-professional APCs include a variety of cell types, such as epithelial cells, fibroblasts, and certain immune cells, among others, which may exhibit different functions in different pathological environments. For example, AECs may play different roles in healthy and diseased states, while their specific role in the immune response is not fully understood. Therefore, how to precisely identify and classify these cells, and how to assess their function in specific diseases, is a major challenge. The function of lay APCs is often spatio-temporal specific and may change with disease progression. These cells may exhibit different immunomodulatory effects in different immune environments in different lung diseases. How to identify and regulate the function of these cells, especially at different disease stages, remains a major challenge in translational medicine. The complex interactions between non-professional APCs and immune cells, especially the regulation of responsiveness by T cells, B cells, and macrophages, are not fully understood. How to regulate the interactions of these cells to achieve immune tolerance or immune activation and avoid excessive immune responses is a major problem in clinical applications.

In conclusion, studying non-professional APCs in lung diseases reveals their crucial role in immune regulation and opens new avenues for clinical applications.

## 5 Key issues and highlights of future research on non-professional APCs

In the future, research on non-professional APCs will focus on several key issues and highlights. Addressing these will enhance our understanding of non-professional APCs in immune regulation and disease, and support their clinical applications.

Firstly, exploring the functional heterogeneity of non-professional APCs in various disease microenvironments is crucial. Non-professional APCs, such as AECs, ECs, and fibroblasts, exhibit diverse functions in diseases like COPD, IPF, and asthma. Their activation status and antigen presentation abilities are influenced by inflammatory factors, metabolic signals, and interactions with other immune cells. Future research must investigate how these cells behave in different pathological contexts. Secondly, the interaction between non-professional APCs and professional APCs warrants further study. Professional APCs, such as DCs and MACs, and non-professional APCs often work together in immune responses. In certain lung diseases, non-professional APCs may collaborate with professional APCs to regulate antigen presentation and T cell activation. Understanding these interactions is vital for unraveling complex immune networks, especially in chronic inflammatory and fibrotic conditions. Future studies should focus on elucidating these mechanisms.

Additionally, examining the dynamic changes of non-professional APCs in the immune microenvironment is important. These cells show functional changes throughout different disease stages, transitioning from a resting to an activated state, which can influence disease progression. This dynamic behavior reflects the plasticity of non-professional APCs. Advanced technologies, such as single-cell sequencing, time series analysis, and spatial omics, can map these changes across various pathological stages. This approach will clarify the specific roles of these cells in disease progression and inform targeted interventions. Furthermore, regulating MHC II expression on non-professional APCs could provide new strategies for vaccine development and immunotherapy. By enhancing MHC II expression, researchers could improve vaccine efficacy and design more effective strategies by targeting specific signaling pathways in these cells. Finally, emerging technologies will play a significant role in future research on non-professional APCs. Innovations such as single-cell multi-omics, spatial omics, mass spectrometry, and advanced flow cytometry will provide higher resolution and broader analysis of non-professional APCs functions. Additionally, new models like *in vitro* organoids and microfluidic chips will offer intuitive platforms for studying these cells in complex environments. These technologies will deepen our understanding of non-professional APCs and enhance their application in disease models, supporting their clinical translation.

## 6 Conclusions and prospects

**Figure 2:**
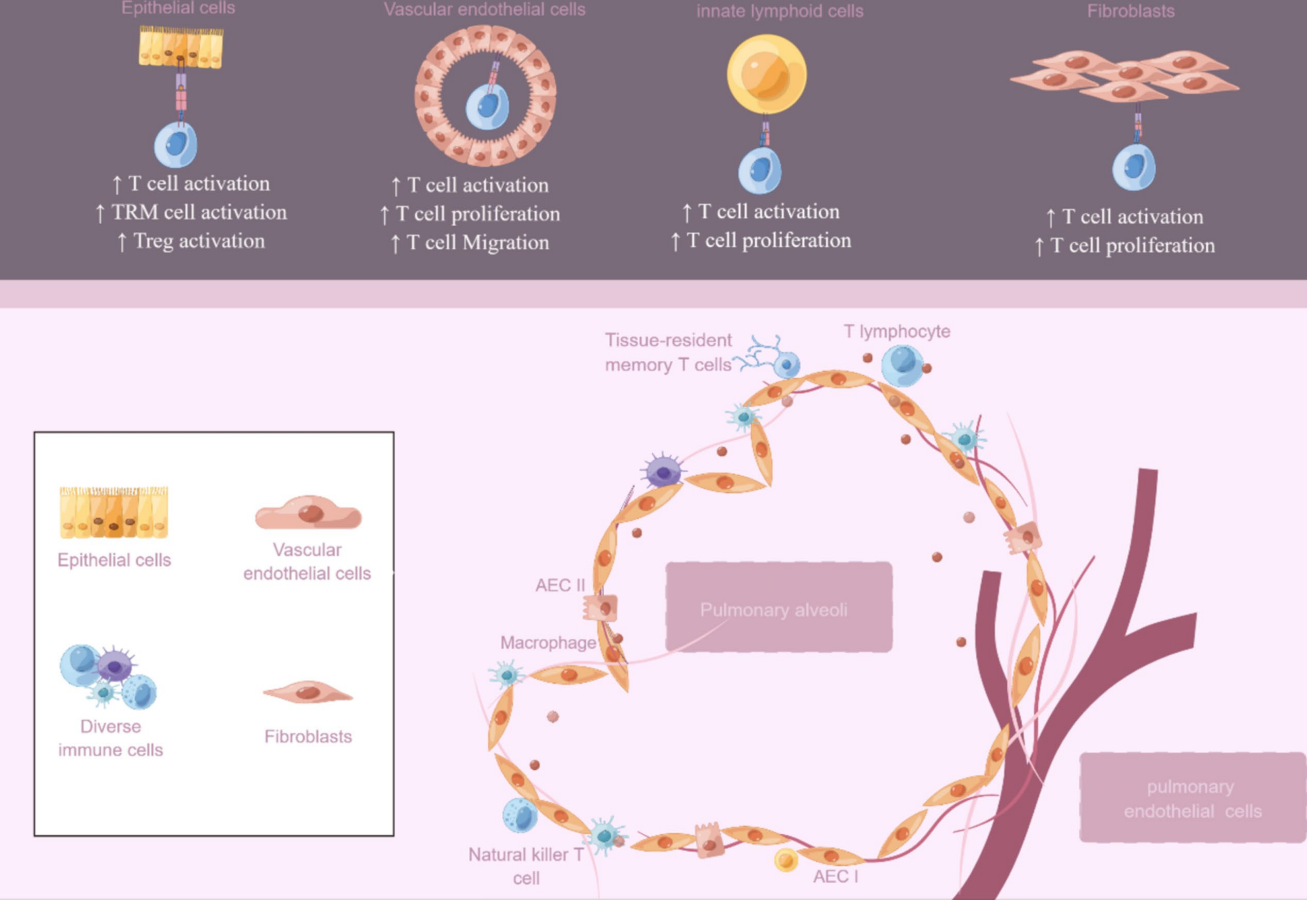
Non-professional antigen-presenting cells in the lung. An overview of the potential roles of non-professional APCs includes epithelial cells, vascular endothelial cells, innate lymphoid cells, and fibroblasts. The figure was created using Figdraw (www.figdraw.com).

In conclusion, although non-professional APCs do not play a dominant role in the immune response as professional APCs such as DCs, they can promote the proliferation and differentiation of T cells by presenting antigen, secreting cytokines, and expressing costimulatory molecules, and play an important auxiliary role in the immune response, especially in local immune and inflammatory reactions. To summarize, we have organized the documented non-professional lung APCs and their function in T cell reactions across various lung illnesses (Table 2; Figure 2). Gaining insight into the practical significance of non-professional APCs in presenting MHC II antigens in various situations could offer a basis for selectively adjusting this process to boost immune protection or alleviate harmful inflammation. Nevertheless, numerous domains necessitate additional examination, and the antigens they handle and exhibit *in vivo* under diverse circumstances remain mostly unidentified. These findings could potentially offer crucial insights into whether MHC II antigen presentation is employed to bolster the inflammatory response of anti-pathogen T cells or to facilitate tissue healing procedures. After viral damage, the diversity of the interaction between local T cells and both professional and non-professional APCs increases. The activation of memory T cells from various subsets of APCs may extend protective immune responses, allowing for the possibility of functional immune responses. The specific involvement of non-professional APCs in the display of MHC II antigens and the indirect control of T cell reactions through local means remains uncertain. Moreover, the unclear regulation of non-professional APCs expression involves the intricate interaction between microbiota, professional APC, and T cell responses.

**Table 2:** Pulmonary non-professional APCs and their roles in T cell responses (current evidence suggests that non-professional APCs play an antigen-presenting role in respiratory diseases, including the expression of MHC complex genes and functional demonstration of antigen presentation in human and animal studies).

Disease	APCs cell type	T cell type	Expression of MHC II machinery	Expression of costimulatory molecules (B7-1/CD80 or B7-2/CD86)	Outcome	References
Influenza virus	Epithelial cell	CD4^+^T	MHC II↑	Not tested	Improvement in outcomes of respiratory viral diseases	Toulmin et al. (25)
*Streptococcus pneumoniae*	Epithelial cell	CD4^+^TRM	MHC II↑	CD86↑	Barrier immune homeostasis	Shenoy et al. (33)
Chronic T cell-mediated pulmonary inflammation	Epithelial cell	CD4^+^T	MHC II↑	Not tested	Autoimmune tissue suppression and immune self-tolerance restoration	Gereke et al. (34)
NTHi bacteria	Fibroblasts	CD4^+^T	MHC II↑	Not tested	Bacterial infection resistance	Hutton et al. (49)
Human lung non-small cell carcinomas	Antigen-presenting cancer-associated fibroblasts	CD4^+^T	MHC II↑	Not tested	Tumor suppression	Kerdidani et al. (26)
Acute exacerbation of chronic obstructive pulmonary disease	ILC2s	CD4^+^T	MHC II↑	CD80↑	Promotion of Th2 cell differentiation	Jiang et al. (53)
RSV	ILC2s	CD4^+^T	MHC II↑	CD80, CD86↑	Airway inflammation induction	Kang et al. (54)
RSV infection induces an asthmatic response	Eosinophils	CD4^+^T	MHC II↑	CD80, CD86↑	Exacerbation of inflammatory and allergic reactions	Zhang et al. (62)
There was no specific medical background	Mast cells	CD4^+^T	MHC II↑	Not tested	Induce the differentiation of naive CD4^+^T cells toward conventional Th2 cells	Nakano et al. (63)

The concept of non-hematopoietic cells playing a significant role in the initiation of the pulmonary immune response is currently under investigation. Nevertheless, our knowledge regarding the precise role of pulmonary non-professional APCs is restricted. One reason for this is due to various significant assumptions, including the observation that MHC I and II levels are increased in numerous illnesses. Nevertheless, these modifications by themselves do not ensure particular immune responses to antigens. Exposure to an inflammatory setting can alter the upregulation of MHC I and II expression. Nonetheless, the establishment of effective immune synapses between APCs and T cells necessitates supplementary costimulatory molecules, alongside the existence of MHC I and II machinery, and ultimately results in the activation of T cells (19). Hence, additional research is required to ascertain the impact of selective absence of MHC I and II presentation in non-professional APCs, like respiratory epithelial cells. This investigation aims to explore the influence of MHC II expression in non-professional APCs on local immune balance, addressing both APC-related inquiries and those unrelated to APCs function, thereby enhancing our comprehension of the role played by context and magnitude of MHC II expression in non-professional APCs.

In summary, future research on non-professional APCs will address functional heterogeneity, cell interactions, dynamic regulation, clinical translation challenges, and emerging technologies. Progress in these areas will establish a comprehensive understanding of non-professional APCs’ roles in immune regulation and disease, paving the way for their use in immunotherapy.
